# Unique attributes of official endorsers in destination marketing

**DOI:** 10.1038/s41598-024-64951-3

**Published:** 2024-06-17

**Authors:** Yipeng Zhao, Yan Li, Bo Liu, Haodong Chang, Yining Guo

**Affiliations:** 1 College of Management Science, Chengdu University of Technology, Chengdu, China; 2 College of Mathematics and Physics, Chengdu University of Technology, Chengdu, China

**Keywords:** Official endorsements, Endorsement traits difference, Identification theory, Structure equation model, Human behaviour, Psychology and behaviour

## Abstract

Along with the digital transformation of the administrative environment and the end of the COVID-19 pandemic, official endorsers have nurtured a new channel for tourism destination marketing, which is of great significance to local economic recovery. However, less attention has been paid to the different effects of endorsement between ordinary endorsers and official endorsers, mainly due to their contrasting social statuses. To bridge the research gap, the source credibility model and social identity theory are integrated to construct the distinctive attributes of officials, as well as structural equation model is utilized to explore the underlying mechanism of official endorsement. Findings indicate that trustworthiness, the sense of authority, expertise, and attractiveness have direct positive effects on official identification, while also indirectly influencing tourists’ attitudes toward the destination through official identification. These findings provide theoretical and managerial implications for the local government managers involved in tourism destination marketing.

## Introduction

Official endorsements for marketing tourist destinations have emerged as a new phenomenon in China after the end of the COVID-19 pandemic. This emerging trend presents a distinct and promising approach to promoting tourist destinations, which is similar to the practice of celebrity endorsements. Celebrity endorsements, which have been employed as a marketing strategy since the late nineteenth century^1,2^, were initially utilized for brand marketing. Subsequently, this approach has found widespread application in the promotion of products^3^, political candidates^4,5^, and destination marketing^6–8^. Within the tourism sector, celebrity endorsers play a significant role in shaping destination images, which in turn influence the destination choices of potential consumers^9^. Thus, celebrity endorsements have boomed in Western and Eastern countries, with notable examples including actor Chris Hemsworth as the face of Tourism Australia and actor Jackie Chan as Asia’s Tourism Ambassador. However, there is a significant difference between official and ordinary celebrities. The difference between official and ordinary celebrities in endorsement is mainly reflected in their social statues, endorsement motivation and social influence. Official endorsers usually endorse tourism destinations related to their scope of responsibilities, so their endorsement behavior has certain authority and formality. Their endorsements are often regarded as government support and recommendation, which can enhance tourists’ sense of trust and security of destinations or products. In contrast, ordinary celebrity endorsements are more influenced by personal preferences, business cooperation or personal image building. Their influence comes mainly from their personal popularity, charisma and fan base. Although they can attract a lot of attention, their endorsements may lack the official recognition and authority of government officials. Therefore, there are significant differences between official and ordinary celebrity endorsements.

In the current era of flourishing digital economy, the administrative environment is undergoing significant digital transformation. Officials, leveraging digital technologies, particularly through platforms like TikTok, are seizing the opportunity to showcase local customs and human characteristics, which aims to captivate both domestic and international tourists and offers them immersive glimpses into the cultural richness and scenic beauty of the region. Notably, Liu Hong and He Jiaolong, officials from the Department of Culture and Tourism in China, have achieved commendable success in their promotional efforts, such as 4.6 billion online exposure and 2.75 million RMB sales in single live stream revenue^10^. Additionally, several studies have explored the characteristics of interaction rituals between government affairs and users within the theoretical framework of interactive ritual chains^11^ such as the symbolization of official identity^12^. Research has also focused on examining the phenomenon of “officials living with goods,” investigating its formation mechanism, practical challenges, and potential future developments^13,14^. Thus, official endorsements seem as a better option for tourism destination marketing than ordinary individuals^15,16^. Essentially, the idea behind celebrity-endorsed destination campaigns is straightforward: capturing the attention of people^7^, enhancing the persuasiveness of the message^17^, and ultimately improving tourism attitude that may translate into practical actions of potential tourists. Numerous studies have demonstrated attitude as a crucial mediator of behavioral intentions^18–20^. Within the realm of tourism, the attitude of tourists holds significant importance as it influences various aspects such as destination selection^21–23^, the cultivation of travel intentions^24,25^, indirect impacts on tourists' future behavior^26^, and the effectiveness of endorsements^27^. Besides, van der Veen^8^ found that attractiveness and expertise positively affected tourists’ attitudes based on the source credibility model suggested by Ohanian^28^. However, previous research has primarily concentrated on unidimensional perceptions, including attractiveness, trustworthiness, and expertise, which leads to neglecting comprehensive evaluations encompassing multiple dimensions^28,29^. Moreover, existing studies posit that official endorsement represents a form of government innovation^30,31^. Centering on public organization innovation and its driving factors, studies have been conducted from the aspects of the external environment, internal organizational characteristics, and leadership traits^32^. Li^33^ argue that official endorsement can be seen as an innovative endeavor undertaken by pioneering leaders from the perspective of policy entrepreneurs, as it not only consumes scarce time and precious attention, but is also closely tied to their innovative character^34^. On the other hand, official endorsement also serves as a means of commercial promotion through livestreaming platforms, scholars have examined how local governments utilize limited resources such as administrative power, human resources, reputation capital, and policy information to facilitate the growth of distinct local industries based on the “governments and firms” theory^35^. Some other studies have highlighted three primary driving factors for rural e-commerce entrepreneurship: the acquaintance society, the online market, and rural talent^36^. Therefore, contrary to ordinary celebrities, official endorsement campaigns can be regarded as altruistic activities that not only serve as a catalyst for the economic recovery of local tourism but also foster innovation within government services. Moreover, the practice of those campaigns contributes to the establishment of a service-oriented government and the exploration of new developments within the digital technology-enabled cultural tourism industry. Consequently, less attention has been paid to the different endorsement effects of official and celebrity endorsements on tourists’ attitudes toward destinations, which leads to several urgent research questions: (1) What are the distinct characteristics of government official endorsers compared to ordinary celebrity endorsers in the context of destination marketing? (2) How do the attributes of trustworthiness, authority, expertise, and attractiveness in official endorsers influence tourists’ attitudes towards destinations? (3) What is the mediating role of official identification in the relationship between the attributes of official endorsers and tourists’ attitudes towards destinations? (4) How can local governments leverage the unique attributes of official endorsers to enhance their destination marketing strategies and support local economic recovery post-COVID-19? Therefore, the objective of this study is to explore the unique characteristics and effectiveness of government official endorsers in destination marketing, which in order to bridge the gap in the literature regarding the differences between ordinary celebrity endorsers and official endorsers in destination marketing.

Since the significant difference between official and ordinary endorsers, this paper examines that whether official endorsers positively affect tourism attitudes and its underlying mechanism. The study addresses the critical need to fill the research gaps regarding the role of government officials as endorsers in destination marketing. By linking these gaps with the research problems, this study aims to provide comprehensive insights and practical solutions to enhance the effectiveness of official endorsements in tourism promotion, ultimately supporting local economic recovery and development. To this end, both conceptual models and research hypotheses are proposed based on the source credibility model and social identity theory. Then, data collection is facilitated through a questionnaire survey, as well as the sample characteristics are analyzed by the SPSS 26.0 software (URL: https://www.ibm.com/support/pages/downloading-ibm-spss-statistics-26). In addition, AMOS 24.0 software (URL: https://www.ibm.com/support/pages/downloading-ibm-spss-amos-24) is utilized to conduct path tests and explore the effect of factors in the conceptual model based on structural equation modeling. According to the analysis of results, reasonable discussion and targeted suggestions are proposed for local government employees, which aim to provide a scientifical basis to blaze a sustainable path in the future.

## Literature review

To comprehensively explore the underlying mechanism of official endorsers, this paper first reviews the mainstream research direction of celebrity endorsement and the research of celebrity endorsement in destination marketing. Moreover, the social identity theory has been reviewed and explained based on four theories. Finally, we review the research on tourist attitudes toward destinations.

### Overview of celebrity endorsements research

Primary research streams have emerged from three perspectives on celebrity endorsements, including the source credibility stream, the meaning transfer stream, and the congruence stream.

Research concerning the source credibility has shown that the perceived credibility of the celebrity endorser is one of the reasons for its effective endorsement. Carl I Hovland^37^ believed that the celebrity’s source reliability includes the professionalism and reliability of the source. In addition to these two dimensions, McGuire^38^ believes that attractiveness cannot be ignored. Ohanian^28^ believes that the three dimensions of expertise, trustworthiness, and attractiveness can be used to measure source credibility. Moreover, the positive effect of all three dimensions has been observed, such as trustworthiness^39,40^; Maddux and expertise^41,42^; attractiveness^43,44^.

The second stream is the meaning transfer model, which originates from McCracken^45^ who conceptualized celebrity endorsements as a process of transferring meaning and considered celebrities to serve as repositories of meaning, which is subsequently transferred to the product through advertising. Then, consumers absorb these meanings through the acquisition and utilization of products and services. Walker^46^ have examined celebrity traits as a surrogate for meaning and have observed their successful transmission to the endorsed product.

The third stream is the congruence hypothesis, which is used to measure endorser effectiveness^47,48^. Research about the endorser and product has shown that higher congruence means stronger endorsement effectiveness. Simultaneously, existing research perspectives have been extended to brand-endorsers’ personalities^49^, age endorses^50^, and country-of-origin-endorsers^51^ congruence.

To sum up, the source credibility model has garnered significant research attention in destination marketing^39–44^. Compared with the other two theories, the source credibility model can more directly reflect the characteristics of official endorsers. Thus, it has been considered as the basic model in this paper.

### Celebrity endorser in destination marketing

The research examining the impact of celebrity endorsers in destination marketing is comparatively limited compared to the extensive literature on the mainstream of celebrity endorsements. Furthermore, the majority of celebrity endorsement research has primarily concentrated on products and services, leading to a relative dearth of research examining the effects of endorsers within the domain of destination marketing^52^. A celebrity endorser is defined as an individual who possesses public recognition and employs this recognition to promote a product via advertisements^45,53,54^. The category of celebrity endorsers has once encompassed a broad range of groups, including movie and television stars, athletes, politicians, business figures, artists, and military personnel^45,55,56^. With the rapid advancement of technology, individuals who have gained fame through social media have also been called "Internet celebrities" due to their substantial social presence, which significantly influences the behaviors of their audience^55^. Extensive research has demonstrated the positive effects of celebrity endorsements on various aspects, such as brand awareness and loyalty^57^, attitudes toward advertising messages^43^, brand attitudes^58^, and purchase intention^59–61^. Within the tourism sector, two perspectives exist regarding the impact of celebrity endorsements on destination marketing. The first perspective primarily focuses on the attributes of celebrity endorsers, such as the sense of belongingness between the brand and endorsers^7^, as well as the influence of celebrity image on destination awareness and purchase decisions^9^. The second perspective examines the diverse active responses to celebrities exhibited by tourists with different demographic characteristics, including factors such as age^62^, income level^63^, and other relevant characteristics^64^.

The utilization of celebrity endorsers to promote destinations has garnered significant attention from researchers. However, there has been a dearth of studies focusing on official endorsers, who differ from ordinary celebrities by their association with destination government support. Thus, the distinct characteristics of official endorsers such as the sense of authority have not been discussed and examined in current research.

### Source credibility model of endorsers

Ohanian^28^ integrated the concepts of credibility and persuasibility^65^ along with attractiveness^38^ to propose a three-dimensional framework for assessing source credibility, including attractiveness, trustworthiness, and expertise. Attractiveness, as defined by Morrow^66^, pertains to the ability of the endorser’s image to elicit favorable responses from others. Trustworthiness reflects the consumers’ perception of the endorser’s honesty, integrity, and believability, while expertise relates to the endorser’s knowledge, skills, and experience^28^. Moreover, improving attractiveness and enhancing credibility have been found to positively influence customer attitudes and behaviors^59,67,68^. In the tourism sector, numerous scholars have examined the role of endorser credibility in destination marketing. For example, studies have shown that the attractiveness of endorsers positively affects individuals’ attitudes toward both the advertisement and the destination itself^7^. Additionally, the perceived trustworthiness and expertise of endorsers have been found to positively influence tourists’ brand affinity toward a destination^56^. Furthermore, it has been identified that expertise is the most significant factor associated with destination attachment^69^.

However, with the economic recovery in the post COVID-19 era, a new trend of official endorsement has emerged in certain regions of China, particularly in the central and western ethnic areas. Prominent examples of this phenomenon include Liu Hong in Sichuan Province and Jiaolong He in the Xinjiang Uygur Autonomous Region, who utilize their official positions to advocate for local customs and cultural traits. Given their unique social status, which sets them apart from ordinary celebrities, it becomes imperative to explore the underlying mechanisms of official endorsement and distinguish between officials and celebrity endorsers.

### Social identity theory

Social identity theory (SIT) posits that an individual’s identity is a cognitive element of their self-concept acquired through group affiliation^70^. SIT further suggests that social identity involves the psychological mechanism of adopting the attitudes and behaviors of groups or others, integrating them into one’s personality. This process of identity influences individuals’ thoughts, emotions, attitudes, and behaviors, aligning them with the objects or entities they identify with Ref.^71^, which can be examined through the lenses of multiple theories, including dramatism theory, the theory of opinion change, social cognitive theory^72^, and parasocial relationships. Dramatism theory contends that the premise of effective communication relies on identifying the audience and establishing a common bond based on similar values and perceptions between the audience and the characters. Consequently, the audience is more likely to be influenced by the performance when this bond is established. The theory of opinion change proposes three processes of social influence: compliance, identification, and internalization. Kelman^73^ argues that identification occurs when individuals accept the attitudes and behaviors of others or groups to establish and maintain satisfactory relationships with them. The social cognitive theory maintains that an individual’s likelihood of performing a certain behavior depends on their identification with a behavioral model, with this identification process influenced by the perceived similarity between the model and the individual. The concept of parasocial relationships asserts that individuals can develop a sense of identification with media celebrities via media channels, as media personalities become familiar figures to the audience.

In general, many researches explain and support social identity theory from several perspectives, As well as the mediating effect of identity on celebrity effect is also examined^74^. However, few studies have explored its effect within the context of official endorsements. To comprehensively understand the underlying official endorsements and the mediating effect of official identification, social identity theory has been considered to construct the conceptual model in this paper.

### Tourist attitude toward the destination

According to Ajzen^75^, attitude refers to the extent to which an individual holds different evaluations for a particular behavior. Moreover, individuals with a more favorable attitude toward a behavior are more inclined to engage in it. In the tourism sector, tourists' attitudes toward a destination serve as indicators of their evaluation of the location, which is considered as a critical factor influencing tourists’ travel decisions^76^. In terms of the determinants of tourist attitude, extensive research has been conducted from various perspectives, including studies focused on tourists themselves, destinations, and other relevant perspectives. Researchers have explored factors such as the image of the destination^25^, electronic word of mouth^76^, and source credibility^7^. Additionally, tourists’ motivation^77^, beliefs^78^, and perceived value^79^ have been identified as significant factors for tourist attitudes towards destinations.

In summary, celebrity endorsements usually do not directly affect individuals’ behavioral intentions^42,80^. Moreover, researchers have demonstrated that attitude is a significant mediator factor to affect individuals’ behavioral intentions^18–20^. Thus, it is necessary to study tourist attitudes toward destinations within the context of official endorsement.

## Hypothesis development

### Credibility and social identity theory

In the celebrity endorsers research, the source credibility model has received support in the research of the effects of celebrity endorsements, such as in attractiveness^7,43,44,81^, trustworthiness^39,40,56^, expertise^41,42,69^. Besides, research has consistently shown that the characteristics of endorsers significantly influence consumer attitudes and perceptions. Choi and Rifon found that attractive endorsers are more likely to create a favorable impression and increase the likability of the promoted destination^48^ and consumer perceptions and attitudes^61^. Trustworthiness is another crucial factor. Trust in the endorser enhances the perceived reliability of the information, leading to a positive attitude towards the destination^82^. Expertise of the endorser also plays a vital role to influence attitudes and behaviors of the audience^83^ and consumers' attitudes towards products and destinations^61^. Additionally, authority of the endorser significantly impacts persuasion, impacting attitudes and behaviors^37^. Endorsements by authoritative figures can lead to higher trust and acceptance of the message^84^. These findings collectively underscore the importance of endorser characteristics in shaping consumer attitudes and highlight the multifaceted nature of effective endorsements. As mentioned above, there are differences between officials and celebrities. Due to the relatively strict selection process of Chinese government officials, the Chinese government generally has high credibility in social management and public services, which leading the natural authority of the status of officials in the minds of the masses. It is a commendable and excellent behavior that government officials with authoritative status can help the destination’s publicity and marketing, which will lead to personal admiration^85,86^. Additionally, due to cultural influence, China has a higher power index^87^. People with higher social status are more likely to obtain social recognition in the group^88^. Thus, the following hypothesis was developed:

#### H1

Official endorser’s attractiveness has positive effects on official identification.

#### H2

Official endorser’s trustworthiness has positive effects on official identification.

#### H3

Official endorser’s expertise has positive effects on official identification.

#### H4

Official endorser’s authority has positive effects on official identification.

### Social identity theory and tourist attitude

According to social identity theory, the mental process of imitating the attitudes of others induces to changes in their attitudes and makes them consistent with the identified objects. Moreover, the social cognitive theory holds that the possibility of an individual performing a certain behavior depends on his identification with the behavior model, and this identification process depends on the perceived similarity between the behavior model and themselves^72^. Previous scholars found that identity does have a significant impact on people’s attitudes and behaviors, as well as the higher the degree of recognition of a celebrity, the higher the possibility that people are engaged in the behavior advocated and demonstrated by the celebrity^74,89–91^. Ashforth and Mael^92^ indicates that when individuals identify with a group, they are more likely to adopt attitudes and behaviors that favor the group and its recommendations. Thus, the following hypothesis was developed:

#### H5

Tourists’ identification with the official positively influences their attitude towards the destination endorsed by the official.

### The mediating role of official identification

Prior research have explained social identity theory from four perspectives including dramatism theory, the theory of opinion change^73^, social cognitive theory^72^, and parasocial relationships^93^. Research has demonstrated the significant impact of identification on tourists’ intentions. Kim and Stepchenkova^94^ explored how celebrity endorsements affect destination image and found that identification with the endorser critically mediates tourists’ perceptions and attitudes towards destinations. Similarly, official endorsements, particularly by high-ranking government officials, foster a strong sense of identification among tourists, thereby positively influencing their attitudes towards the destination^95^. Qu, Kim, and Im^96^ also found that tourists who identified with the values and image of the endorsing official were more likely to develop positive attitudes towards the endorsed destination, further supporting the mediating role of official identification. These findings collectively underscore the importance of endorser identification in shaping tourists' attitudes and highlight the distinct impact of both celebrity and official endorsements on tourism outcomes. In addition, Basil^74^ examines the mediating role of identity in celebrity and finds that celebrity identity can lead to sustained attitude and behavior change. The mediating effect of celebrity identification is examined between the perceived celebrity image and the attitude toward the destination^97^. Moreover, Li Liu^98^ find found the mediating role of celebrity identification in self-congruity and attitude toward destinations. Thus, to test this mediating mechanism, the following hypothesis was proposed:

H6: Official identification mediates the effect of official characteristics on tourism attitude toward the destination.

**Figure 1:**
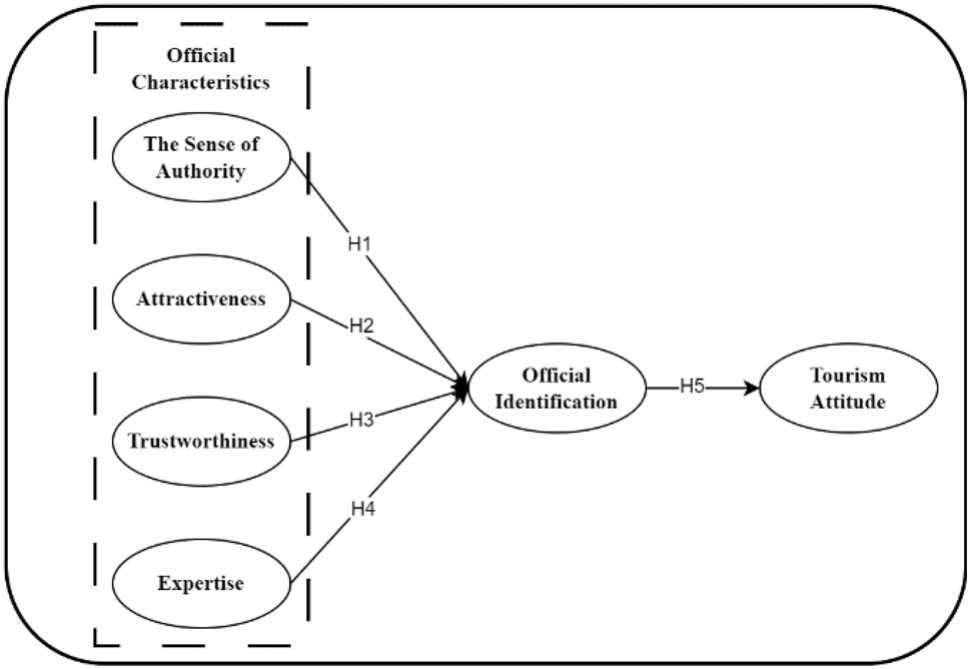
Conceptual model of official endorsers.

To sum up, the research framework of this paper is shown in Fig. 1.

## Research methodology and data

Positivist paradigms and quantitative research had been conducted to verify proposed hypotheses. An online survey was designed and a quota sampling technique was used to gather samples, which involves benefits such as no geographic restrictions, lower expenses, and short time consumption. Then, the collected data was analyzed by CB-SEM methods.

### Data collection and sample profile

A total of 75 questionnaires were collected in the pre-survey, and based on passing the reliability and validity test, questionnaire links were distributed through Questionnaire Star (a questionnaire survey platform in China) from October 31 to November 25, 2022. First, to define potential tourists and improve the accuracy of the research results, the following two questions will be answered before the respondents fill in the questionnaire. Are you planning to travel within the next 1 year? Have you traveled in the past year? At least one of the two answers chooses “yes”, which is tentatively designated as a potential tourist in this study. Then, all participants received prior notification regarding the research’s objectives and the voluntary nature of their participation before being invited to complete an online questionnaire. Ample time was provided with to complete the questionnaire in their native language, Chinese. Further, participants were explicitly informed that their responses would remain strictly confidential, and they were instructed to submit their answers only if they were familiar with the official endorser’s actions and this experiment has received informed consent from all participants. Additionally, before completing the questionnaire, the survey administrators presented a concise video for introducing. Finally, data was generated from 260 individuals and 241 valid questionnaires were obtained, which is reasonable sample size for SEM analysis^99^. As seen from Table 1, there was an almost equal distribution between males and females. Ages from 18 to 25 were the largest group in the sample, followed by the age group of 26–40, which means that the sample is concentrated on young people. This is consistent with the conclusion of the “China Economic Life Survey” (China’s largest media livelihood survey activity, jointly founded by CCTV Financial channel, the National Bureau of Statistics, and China Post Group), that is, the post-1995 generation has become the main force of tourism consumption.

**Table 1:** Pain points of digital transformation.

Variable		Numbers	Percent
Gender	Male	130	54%
	Female	111	46%
Age	Below 18	34	14.1%
	18–25	152	63.3%
	26–45	45	18.5%
	Over 46	10	4.1%
Qualification	Intermediater	56	23.2%
	Undergraduate	145	60.2%
	Postgraduate	38	15.8%
	PhD	2	0.8%
Occupation	Students	98	40.7%
	Salaried individuals	85	35.3%
	Businessman	43	17.8%
	Retired	15	6.2%

### Survey instrument

Followed the procedures of Churchill Jr^100^, variables items were originally developed based on extant literature and then translated to Chinese for participants by the researchers. Moreover, a bilingual speaker (Chinese and English) and several management and tourism professors scrutinized to ensure the translation accuracy of the original items and the content validity of the current items. The conceptual model included six constructs, among them, the sense of authority, trustworthiness, attractiveness, and expertise were adopted to assess the characteristics of official endorsers adapted from Ohanian^28^ and Zhu and Chang^31^. Similarly, official identification were measured by 3-items derived from Basil^74^; Mael and Ashforth^101^; Martínez and Del Bosque^90^. Besides, a 3-items scale of the tourism attitude was adapted from Ajzen^102^. Finally, all items within the conceptual model were assessed on a 5-point Likert-type scale, with reply categories ranging from 1 = strongly disagree to 5 = strongly agree (Table 2).

**Table 2:** Pain points of digital transformation.

Constructs and indicators	Items	Std	Cronbach’s a	CR	AVE
The sense of authority (SA)	SA1	0.688	0.811	0.816	0.528
	SA2	0.666			
	SA3	0.732			
	SA4	0.811			
Attractiveness (AT)	AT4	0.773	0.841	0.841	0.570
	AT3	0.751			
	AT2	0.735			
	AT1	0.761			
Tourism attitude (TA)	TA3	0.774	0.771	0.769	0.528
	TA2	0.641			
	TA1	0.757			
Official identification (OI)	OI3	0.703	0.806	0.817	0.599
	OI2	0.819			
	OI1	0.795			
Expertise (EX)	EX3	0.738	0.782	0.785	0.549
	EX2	0.738			
	EX1	0.746			
Trustworthiness (TR)	TR3	0.861	0.788	0.799	0.573
	TR2	0.746			
	TR1	0.650			

*CR* composite reliability, *AVE* average variance extracted.

### The method of data analysis

To comprehensively explore the underlying mechanism of official endorsers, a combined method of structure equation model and fuzzy set qualitative comparative analysis was employed to verify the proposed hypotheses. CB-SEM is more suitable to theory testing and confirmation. Thus, CB-SEM technique was adopted to explore the effect of official endorsements in this paper following the suggestions of Dash^103^.

### Ethical approval

This study was approved by the ethnics committee of Chengdu university of technology. We certify that the study was performed in accordance with the 1964 declaration of HELSINKI and later amendments.

### Consent to participate

Written informed consent was obtained from all the participants prior to the enrollment of this study.

## Data analysis and results

### Data normalcy and common method bias

Before performing the SEM, the normality and common method bias need to be examined to ensure appropriateness and application. The skewness and kurtosis values show no significant problems with the normal distribution. Furthermore, when the sample size is high, small deviations from normalcy are expected in social science research and are not seen as detrimental^104^. In addition, common method bias (CMB) may bias results^105^. Thus, Harman's single-factor score is utilized to assess CMB. The results of the single-factor test indicated that six factors with five values greater than 1 were identified, accounting for 67.038% of the total variance. The variance of the first factor was 39.277%, which did not exceed half of the total variance^106^. Therefore, the study was deemed to be free of significant CMB.

### Measurement model

Following the two-step technique proposed by Anderson and Gerbing^107^, confirmatory factor analysis was initially rated utilizing AMOS 24.0 software with the maximum likelihood method. Several measures of fit were employed to evaluate the fit degree of the measurement model. According to Brown^108^, chi-square is very sensitive to sample size, as well as other fit indices, such as the Tucker-Lewis index (TLI), the comparative fit index (CFI), the root mean square error of approximation (RMSEA), and the standardized root mean square residual (SRMR), should be used to evaluate the overall fit of a CFA solution. Higher values on TLI and CFI indicate that the models are well-fitting^109,110^. On the other hand, RMSEA and SRMR values less than 0.07 imply a strong fit, while values near 0.10 indicate a medium fit^111^. Such indices were chosen for this study because of their generally acceptable performance^109,110^. As shown in Table 3, the CFA model fit the sample data well: χ^2^/df = 2.172, IFI = 0.918, CFI = 0.917, SRMR = 0.056 and RMSEA = 0.063.

**Table 3:** Fit indices of measurement and structural model.

Model-fit statistic	χ^2^/df	SRMR	RMSEA	GFI	AGFI	IFI	CFI	TLI
Recommended	< 3	< 0.08	< 0.08	> 0.9	> 0.9	> 0.9	> 0.9	> 0.9
Measurement model	2.172	0.0558	0.0625	0.877	0.837	0.918	0.917	0.9
Structural model	2.094	0.0558	0.068	0.884	0.843	0.925	0.924	0.907

The reliability and validity of the developed factors were evaluated after the measurement model was established. Thus, both convergent and discriminant validity were evaluated in this paper. The composite reliability (CR) estimate and average variance extracted (AVE) of each variable are used to assess reliability, as well as the CR and AVE values should be at least 0.70 and 0.50. In addition, a structure is regarded as reliable when its loading is more than 0.50. In a measurement model^111^. As shown in Table 2, composite reliability scores ranged from 0.77 to 0.84, while AVE scores ranged from 0.53 to 0.60, both of which were higher than the specified cut-off values of 0.70 and 0.50. These results demonstrate that the measurement model was both mutually valid and trustworthy.

Convergent validity evaluates whether the items of each variable converge with the latent construct, which was assessed using factor loadings, Z-values, and the average variance extracted (AVE)^107^. As presented in Table 4, the factor loadings for each variable were greater than 0.50 (range from 0.64 to 0.86) and significant (p < 0.001). Furthermore, all factor loadings were statistically significant (p < 0.001), with Z-values ranging from 8.93 to 11.64 (exceeding the 1.96 criterion). Moreover, all AVEs above a crucial value of 0.50, indicating that the constructs' convergent validity was demonstrated by Fornell and Larcker^112^.

**Table 4:** Correlation matrix.

Constructs	EX	TR	AT	SA	OI	TA
Expertise	**0.741**					
Trustworthiness	0.340	**0.757**				
Attractiveness	0.362	0.664	**0.755**			
The sense of authority	0.431	0.446	0.605	**0.726**		
Official identification	0.569	0.721	0.737	0.715	**0.774**	
Tourism attitude	0.412	0.522	0.534	0.517	0.724	**0.726**

The bold diagonal elements are the square roots of each AVE.

Discriminant validity measures the correlation between different latent variables in this model. The variables’ square root AVE should exceed the other variables in this model^113^. The diagonal elements, the square root of AVE, present the variance between the variable and items, while the correlation between variables is shown with the off-diagonal elements. As seen from Table 3, the bold square root of AVE values is greater than the correlation between variables, which indicates that all variables in this model met the standard of the discriminant validity and further are suitable for testing the structural mode.

### Structure model

**Figure 2:**
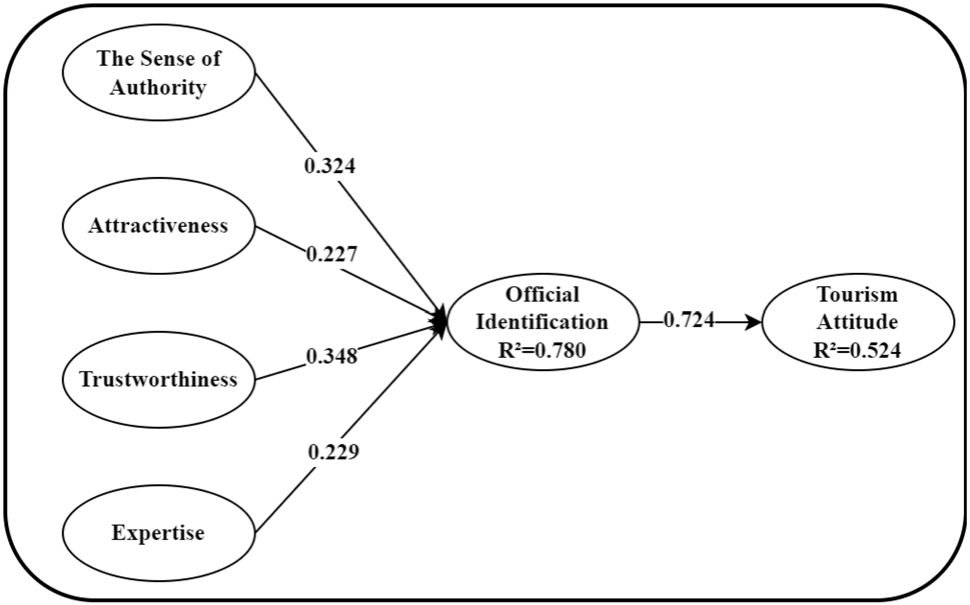
Fitting result of conceptual model.

The measurement model demonstrated a good fit in terms of validity and reliability. Thus, a structural model was employed to test and validate the hypotheses. As seen from Table 3, the data indicated a favorable fit for the structural model, with the following statistics: χ^2^/df = 2.10, p < 0.001, TLI = 0.907, CFI = 0.924, RMSEA = 0.068, and SRMR = 0.0558. Notably, the model accounted for a significant 52.4% of the variance in behavioral intention, suggesting that the conceptual model linking attitudes to behavioral intention is robust in both theoretical and empirical evidence. Furthermore, all proposed hypotheses were supported, as revealed in Table 5 and Fig. 2. The mediating effects related to the hypotheses testing are presented below.

**Table 5:** Estimated standardized coefficients.

Hypothesized relationship	Unstd	S.E	C.R	P	Std	Results
H1: The sense of authority → Official identification	0.402	0.096	4.185	***	0.324	Accept
H2: Attractiveness → Official identification	0.214	0.083	2.583	**	0.227	Accept
H3: Trustworthiness → Official identification	0.311	0.072	4.339	***	0.348	Accept
H4: Expertise → Official identification	0.228	0.063	3.642	***	0.229	Accept
H5: Official identification → tourism attitude	0.721	0.087	8.303	***	0.724	Accept

**p < 0.01; ***p < 0.001.

### Mediation analysis

The mediating effects of celebrity identification were assessed using the bootstrapping method (bootstrap = 1000), which is considered superior to other methods, such as the distribution of product method and Sobel test, for testing mediating effects^114^. In this study, percentile bootstrapping and bias-corrected percentile bootstrapping were performed at a 99% confidence interval, utilizing 10,000 bootstrap samples^115^. Consistent with the recommendations of Preacher^114^, the confidence interval of the lower and upper bounds was calculated to determine the significance of the indirect effects. Table 6 presents the results of the bootstrap test, which confirm a positive and significant mediating effect of celebrity identification between official authority, attractiveness, credibility, expertise, and tourism attitude. Specifically, the mediating effects through authority, attractiveness, credibility, and expertise were 0.29, 0.155, 0.224, and 0.164, respectively, accounting for 34.8%, 18.6%, 26.9%, and 19.7% of the total mediating effects. Further discussion on the hypotheses testing is provided below.

**Table 6:** Mediating effect.

Path relationship	Point estimate	Product of coefficient		Bootstrapping 1000 times 95% CI			
				Bias-corrected		Percentile	
		SE	Z	Lower	Upper	Lower	Upper
Indirect effect							
SA → OI → TA	0.290	0.102	2.843	0.113	0.523	0.101	0.500
ATT → OI → TA	0.155	0.089	1.742	0.002	0.348	0.001	0.347
TR → OI → TA	0.224	0.075	2.987	0.096	0.395	0.081	0.374
EX → OI → TA	0.164	0.062	2.645	0.054	0.302	0.046	0.287
SEIE + TAIE + TRIE + EXIE	0.833	0.092	9.054	0.653	1.020	0.647	1.009
Contrast							
SA vs. ATT	0.136	0.168	0.810	− 0.171	0.490	− 0.207	0.457
SA vs. TR	0.066	0.126	0.524	− 0.166	0.325	− 0.175	0.316
SA vs. EX	0.126	0.119	1.059	− 0.105	0.363	− 0.109	0.351
TA vs. TR	− 0.069	0.142	− 0.486	− 0.350	0.209	− 0.332	0.218
TA vs. EX	− 0.010	0.114	− 0.088	− 0.225	0.215	− 0.210	0.231
TR vs. EX	0.060	0.107	0.561	− 0.147	0.281	− 0.151	0.271
Proportion							
SAIE/TIE	0.348	0.104	3.346	0.149	0.557	0.132	0.541
TAIE/TIE	0.186	0.113	1.646	0.000	0.431	0.002	0.433
TRIE/TIE	0.269	0.090	2.989	0.103	0.454	0.100	0.447
EXIE/TIE	0.197	0.069	2.855	0.063	0.331	0.058	0.325

## Discussion and implications

### Discussion

Our study explores the official endorsement-tourism attitude relationship by highlighting the sense of official authority as a unique characteristic of official endorses, which has rarely been looked at in previous studies but significantly boasts an avenue for destination marketing. In particular, this paper has contributed to advancing previous research by investigating the effects of the sense of official authority, source attractiveness, expertise, and trustworthiness on tourist attitude under the framework of the source credibility model and social identity theory. Moreover, we investigated that official identification has a mediating effect between official endorsers’ characters and tourism attitudes.

Official trustworthiness was observed to be the greatest effect on official identification (βH3 = 0.348, ρ < 0.001), which is in line with the result of Filieri^116^ and Martínez-López^117^. They suggested that if a person is perceived the more honest and sincere, the viewer will receive relevant and informative. Thus, trustworthiness sources are likely to be more influential in social identity among people.

The sense of authority had the second greatest effect on official identification (βH1 = 0.324, ρ < 0.001), which indicated that identification is influenced by the sense of authority. This finding is in line with studies of Solomon^88^ and Flynn^118^ which linked social status to social identification and suggested that people with high social status are more likely to gain social acceptance in a group. Additionally, this paper also confirms the findings of Zhu^31^ research found the sense of identity authority as an important antecedent variable to capture consumers’ purchase intention.

The positive relationship between expertise and official identification had also been verified (βH4 = 0.229, ρ < 0.001). Our results indicated that official identification is likely to be improved when the official endorsements perceived to be expert. This finding is in line with McCormick^119^, Ohanian^42^, and Maddux^41^, which suggested that a well-known endorser with more persuasion and identification.

Celebrity attractiveness had positive impact on official identification (βH2 = 0.227, ρ < 0.01), which suggests that physical attraction is a powerful source to capture attention from people. That is in line with Jeng^120^ and Filieri^116^ studies which found that attractive sources as a prominent predictor of eliciting more favorable attitudes and stronger purchase intentions from participants.

In conclusion, the study’s findings elucidate the complex dynamics of how official endorsements affect tourism attitudes, addressing the core research problem and answering the research questions comprehensively. The integration of Social Identity Theory and the Source Credibility Model has proven effective in capturing these dynamics, providing valuable insights for tourism marketing strategies. This enhanced understanding can guide future promotional efforts, ensuring that endorsements are not only credible and attractive but also capable of fostering strong identification and positive attitudes among potential tourists.

### Contributions

This paper provides both theoretical and practical implications for government managers. Considerable research have been devoted to investigating the influence of celebrity endorsement on tourist attitude based on various model, rather less attention has been paid to official endorsement, a new phenomenon rising in China after COVID-19. Thus, this paper went one step ahead to integrate the sense of authority of official endorsers into the source credibility and attractiveness model as antecedents to examine their impact.

1. This paper selects a fresh research object, namely, government official endorsers. Due to unique official backgrounds, official status naturally possesses the sense of authority for the masses, which is different from ordinary celebrity endorsements and less attention has been paid to the difference between ordinary celebrities vs. official celebrity. Thus, this paper proposes a sense of authority which aim to differentiate the characteristics between ordinary and official endorsers. Subsequently, this paper illustrates that credibility, the sense of authority, expertise, and attractiveness positively influence official identification and tourist attitude based on the source credibility model, source attractiveness model, and social identity theory. Thus, we examine the effectiveness of official endorsements and contribute to extending the scope of application of those models and theories by suggesting that tourism attitudes can be created through official endorsements.
2. To better understand the official endorsement of tourist attitude, the mediating effect of official identification was examined. As well as few studies explored the mediating effect in the context of official endorsement. Specifically, this paper demonstrates the mediating effect of official identification and further finds that trustworthiness and the sense of authority account for more than half of the mediating effect, 34.8% and 26.9% respectively. Thus, the effect of the sense of authority on improving tourism attitudes toward destinations cannot be ignored. Therefore, this paper contributes to extending the source credibility and social identity theory literature by providing a comprehensive view of the effect of official identification.

### Suggestions

1. Due to the greatest directly positive effect on identification and indirectly influence on tourism attitude, much attention thus needs to be paid to the trustworthiness and avoid data fraud. Some officials engage in deceptive practices, such as homogenization and even malicious competition behavior of fake traffic and data, driven by the pursuit of political accomplishments and personal recognition. Thus, institutional guidance and standardized mechanisms for assurance must be proposed. In addition, the primary responsibilities of local governments encompass fostering local economic growth. Therefore, while encouraging the officials actively explore the path of "flow realization" after COVID-19, it is necessary to regulate the network behavior of officials in the system, and establish safeguard measures and fault tolerance mechanisms to ensure that the officials’ behavior is standardized.
2. In light of the direct effect of identification and the greatest indirect influence on tourism attitude, we should prioritize the sense of authority and refrain from embellishing promotion, as excessive propaganda may lead to undermining the credibility of local governments. Thus, local governments must not only rely on officials for online promotion but also emphasize improving infrastructure and public service facilities. These endeavors aim to enrich the tourist experience while consistently improving the foundation in areas such as security, service quality, and integrity management, ensuring effective implementation. Moreover, the availability of high-quality supporting facilities and comprehensive service standards is the cornerstone for the sustainable development of tourist destinations. Thus, governments should concentrate on optimizing service provision quality, enhancing the cultural tourism industry, and establishing standards and norms for the cultural tourism sector. Additionally, it is essential to implement measures that safeguard rights and interests, offer policy support by local conditions and market demands, construct local cultural tourism industry chains and industrial clusters, and promote the high-quality development of the local culture and tourism domain.
3. Based on the positive effect of expertise on official identification, both the expertise of officials and the cultural heritage of destinations should be enhanced and developed. The initial freshness of the official endorsement is easy to wane for individuals, leading to challenges in sustaining development in the tourism sector. Thus, professional officials and cultural enabling are significant starting points to enhance the core competitiveness of tourism destinations and form differentiated development. In particular, as the official promotion has become increasingly homogenized, government employees should delve deeper into local characteristics, folk customs, and natural scenery, which aims to promote novel forms of cross-border integration, facilitating official interactions, and explore innovative communication methods, while avoiding routine and formalized publicity methods.

To sum up, this study proposes several suggestions regarding official endorsement, which aims to help the local government to make destination marketing strategies, innovate new measures of government service, and optimize the new mechanism of tourism governance after COVID-19 in the digital era.

## Conclusion and future research directions

This paper applies the source credibility model to destination marketing for local government to explore the underlying mechanism of official endorsement. Through the analysis of existing literature, we propose the characteristics of official endorsers and develop a conceptual model consisting of the sense of authority, expertise, trustworthiness, and attractiveness as antecedent variables and official identification as mediating variables to influence the tourist attitude. Our findings revealed that the four characteristics of the official endorsement have both direct and indirect effects on tourism attitudes toward destinations, which is significant for local governments to conduct destination marketing strategy. Therefore, we believe that this paper has opened a window for the government to use digital technology to innovate new measures of cultural tourism government services and optimize new mechanisms of tourism governance in the era of the digital economy. However, in real life, other factors affect the audience's travel attitude, such as destination risk, income level, etc. Future studies can further examine other influencing factors and action mechanisms of destination travel attitudes.

## Data Availability

Raw data supporting the conclusions of this paper will be provided by the authors, Yipeng Zhao if necessary.
